# Acute Effects of an “Energy Drink” on Short-Term Maximal Performance, Reaction Times, Psychological and Physiological Parameters: Insights from a Randomized Double-Blind, Placebo-Controlled, Counterbalanced Crossover Trial

**DOI:** 10.3390/nu11050992

**Published:** 2019-04-30

**Authors:** Hamdi Chtourou, Khaled Trabelsi, Achraf Ammar, Roy Jesse Shephard, Nicola Luigi Bragazzi

**Affiliations:** 1 Activité Physique, Sport et Santé, UR18JS01, Observatoire National du Sport, Tunis 1003, Tunisia; h_chtourou@yahoo.fr; 2 High Institute of Sport and Physical Education, University of Sfax, Sfax 3000, Tunisia; ammar.achraf@ymail.com; 3 UR15JS01: Education, Motricité, Sport et Santé (EM2S), High Institute of Sport and Physical Education, University of Sfax, Sfax 3000, Tunisia; trabelsikhaled@gmail.com; 4 Institute of Sport Sciences, Otto-von-Guericke University, 39104 Magdeburg, Germany; 5 Faculty of Kinesiology and Physical Education, University of Toronto, Toronto, ON M5S 1A1, Canada; royjshep@shaw.ca; 6 Department of Health Sciences (DISSAL), Postgraduate School of Public Health, University of Genoa, 16132 Genoa, Italy

**Keywords:** caffeine, energy drinks, fatigue, mood state, exercise

## Abstract

The current study examined the relationships between the effects of consuming a caffeine-containing “energy drink” upon (i) short-term maximal performance, (ii) reaction times, and (iii) psychological factors (i.e., mood state, ratings of perceived exertion (RPE), and affective load) and on physiological parameters (i.e., blood pressure and blood glucose). A randomized, double-blind, placebo-controlled, counterbalanced crossover design was implemented in this study. Nineteen male physical-education students (age: 21.2 ± 1.2 years; height: 1.76 ± 0.08 m; body-mass: 76.6 ± 12.6 kg) performed two test sessions: after drinking the “Red Bull’ beverage (RB) and after drinking a placebo (PL). One hour after ingestion of each drink, resting blood glucose and blood pressure were measured and the participants completed the Profile of Mood States questionnaire. Then, after a 5-min warm-up, simple visual reaction time and handgrip force were measured, and the 30-s Wingate test was performed. Immediately after these tests, the RPE, blood glucose, and blood pressure were measured, and the affective load was calculated. Differences between treatments were assessed using two-way repeated measures analyses of variance and paired t-tests, as appropriate. Relationships between the test variables were assessed using Bland–Altman correlations. Significant (i) improvements in peak and mean power output, handgrip force, pre- and post-exercise blood glucose, blood pressure, and vigor and (ii) reductions in reaction times, depression, confusion, fatigue, anger, anxiety, RPE, and affective load scores were observed after RB compared to PL. There were significant correlations of (i) physical performances and reaction times with (ii) RPE, affective load, and pre- and post-exercise blood glucose levels. Gains in peak and mean power were significantly correlated with reductions in fatigue, anxiety (peak power only), and anger (mean power only). The reduction of reaction times was significantly correlated with decreases in confusion and anger and with increases in vigor. Handgrip force and reaction times were significantly correlated with pre- and post-exercise blood pressures. We conclude that RB ingestion has a positive effect on physical performance and reaction times. This effect is related to ergogenic responses in both psychological (i.e., RPE, affective load, and mood state) and physiological (i.e., blood glucose and blood pressure) domains.

## 1. Introduction

Energy drinks (EDs) are beverages that typically contain a mixture of caffeine, taurine, herbal extracts (e.g., guarana, yerba mate, ginseng), vitamins (e.g., riboflavin, niacin, vitamin B-6), glucuronolactone, proprietary blends, and amino acids [1].

They can boost energy, improve alertness and promote wakefulness when performing high-intensity physical exercise [2], and, for this reason, they have become one of the substances most commonly consumed by athletes and other practitioners of physical activity. According to Froiland et al. [3], some 72.9% of U.S. college athletes are ED consumers.

Caffeine-containing EDs have been reported as beneficial in many sporting activities, possibly by enhancing motor unit recruitment.

Del Coso et al. [4,5] showed that the ingestion of 3 mg/kg of caffeine in the form of a commercially available ED increased overall running pace and sprint velocities during a rugby sevens competition. In adolescent basketball players, the same dose increased jump performance with no adverse effect on basketball shooting precision [6].

Del Coso et al. [7] noted that such an ED enhanced ball velocity in the spike test, the mean height of squat and countermovement jumps, and performance on the 15-s rebound jump test and the agility T-test. Furthermore, during a simulated game, players performed successful volleyball actions more frequently (24.6% ± 14.3% vs. 34.3% ± 16.5%, *p* < 0.05) with ingestion of the caffeinated ED rather than the placebo (PL) [7].

Although several studies have investigated the effects of ED on aerobic performance, there is as yet only limited and inconclusive data about their impact on short-term maximal performance [8]. Fukuda et al. [9] reported that the ingestion of supplements containing creatine, or caffeine plus amino acids improved the anaerobic running capacity by 10.8%.

In contrast, Hahn et al. [8] saw no beneficial effects of caffeine-containing ED on vertical jumping and repeated sprinting (i.e., measures of mean and peak anaerobic power). Likewise, Gwacham and Wagner [10] observed no ergogenic effect of caffeine-taurine ED on repeated sprinting (i.e., 6 × 35-s with 10-s rest intervals).

Studies of relationships between caffeine-containing EDs and psychological variables are also inconclusive to date; however, some studies have reported positive effects on subjective alertness, mental focus, energy, and fatigue tolerance [8,11,12,13,14].

Alford et al. [11] saw a positive effect on reaction time (a decrease of 88.7 msec). Likewise, Hoffman et al. [12] reported significant improvements in focus (+0.5 arbitrary units, AU) and energy (+0.4 AU) after ingestion of caffeine-containing EDs compared to placebo (PL). Hahn et al. [8] also described a significant reduction of perceived fatigue during repeated sprinting, and Wesnes et al. [15] demonstrated significant improvement in the attentional capacity, vigilance, and numeric and spatial working memory of healthy young adults after ingesting caffeine-containing ED.

However, no significant changes in mood state were seen. In contrast, Petrelli et al. [16] reported significant reductions of anxiety and depression after ingestion of caffeine-containing ED consumption compared to PL.

Physiological responses may also be affected by EDs. Del Coso et al. [17] found that caffeine-containing EDs increased systolic and diastolic blood pressures, although Wesnes et al. [15] did not show any significant change in blood glucose after drinking caffeine-containing EDs.

As yet, it remains unclear whether changes in mood state, blood pressures, and blood glucose levels are related to these ergogenic effects. Thus, the purpose of the present study was to examine relationships between the effects of caffeine-containing ED on (i) short-term maximal performance, (ii) reaction times, and (iii) psychological variables (mood state, rating of perceived exertion (RPE), and affective load) and changes in physiological parameters (i.e., blood pressures and blood glucose levels).

We hypothesized that the ergogenic effects of caffeine-containing EDs on short-term maximal performance and reaction times would be related to positive changes in both psychological factors (mood state, RPE, and affective load) and physiological parameters (blood pressures and blood glucose).

## 2. Materials and Methods

### 2.1. Participants Selection: Inclusion and Exclusion Criteria

The sample size was calculated a priori, using procedures suggested by Beck [18] and the software G*Power [19]. Based on the results of Del Coso et al. [4,5], effect sizes were estimated to be 0.62 (medium effect). To reach the desired statistical power and in order to attribute observed differences to factors other than chance alone, a minimum sample of 18 participants was required. To accommodate a possible drop-out of some participants, we recruited a total of 22 healthy and regularly active physical-education male students from various sports disciplines.

Potential participants were initially screened through telephone interviews based on the following inclusion criteria: (i) 18–40 years of age, (ii) body mass index (BMI) less than 25 kg/m^2^, and iii) being low (<1.5 g/month [20]) and not regular caffeine users.

Exclusion criteria included: i) diagnosis of any chronic metabolic disease such as type 2 diabetes or cardiovascular disease, ii) diagnosis of an auto-immune disease such as rheumatoid arthritis, lupus, or type 1 diabetes, liver disease and iii) the intake of any medications or dietary supplements known to influence blood glucose concentrations or blood pressures.

The study was conducted according to the declaration of Helsinki and the protocol was fully approved (identification code: 8/16) by the review board “Local Committee of the Laboratory of Biochemistry, CHU Habib Bourguiba, Sfax, Tunisia.”

After a thorough explanation of the protocol with responses to all questions, participants signed a written informed consent form.

Subjects were instructed to avoid nicotine, alcohol, dietary supplements, medications, and all other stimulants and to maintain their normal dietary, sleep and physical activity patterns before test sessions.

Caffeine and other caffeinated products (e.g., chocolate, caffeinated gums, caffeine-containing beverages) were avoided for 48 h and food for at least 4 h before testing.

### 2.2. Experimental Design

A randomized double-blind, placebo-controlled, counterbalanced, crossover design was adopted for this study. The randomized order of testing was determined using free online software (www.randomization.com).

Neither staff nor participants were informed about the names of the two drinks, and blinding was strictly maintained by emphasizing to both staff and participants that both drinks adhered to healthy principles and that each drink was advocated by certain sports medicine experts.

Two familiarization sessions were completed before definitive test sessions in order to eliminate any learning effects on physical performance and reaction time measurements. During the second familiarization session, body mass, and height were recorded.

**Figure 1:**
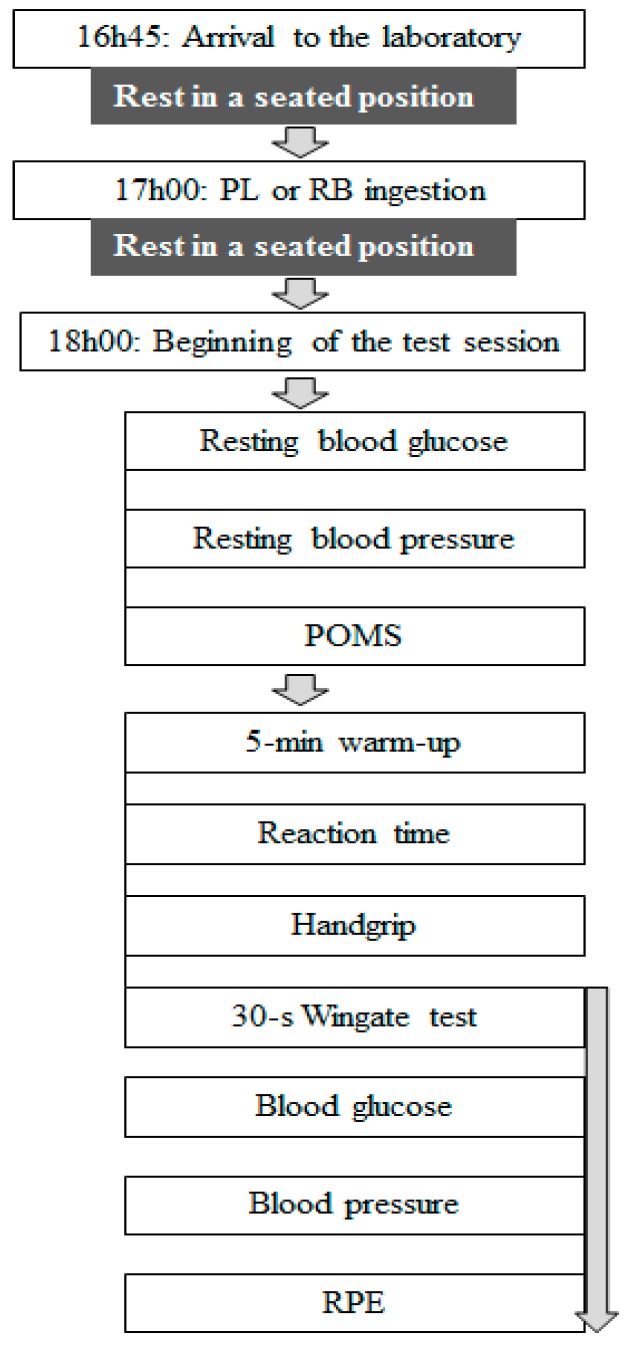
Experimental design. PL = placebo; RB = Red Bull; POMS = profile of mood states; RPE = rating of perceived exertion; HG = handgrip; RT = reaction time.

The experimental design of the present study is pictorially presented in Figure 1.

Each participant visited the laboratory for two formal test sessions, drinking a caffeine-containing ED (RB) and a caffeine and taurine-free beverage drink (PL). All sessions were arranged in the early evening hours to avoid any time of day effects, as suggested by Ammar et al. [21,22,23]. The two definitive test sessions were separated by an interval of seven days to allow sufficient recovery between tests and to ensure caffeine washout. To avoid identification, two opaque and unmarked cans [24,25,26] of RB or PL were ingested by each participant (i.e., 500 mL) in the presence of a researcher. The two drinks were similar in volume, texture, and appearance. One can of RB drink (i.e., 250 mL) contained 80 mg of caffeine, 1 g of taurine, 27 g of carbohydrates, 0.6 g of protein, 5 mg of vitamin B6, and 487 kJ of energy. The PL drink was prepared by an agri-food engineer; it did not contain any caffeine or taurine, but comprised carbonated water, carbohydrates, citric acid lemon juice reconstituted from concentrate (1%), supplemented by flavorings of sodium citrate, acesulfame K, sucralose, potassium sorbate and RB flavoring that contains propylene glycol E1520 (0.23 mL). Of note, both the PL and RB drinks were isocaloric.

Beverages were prepared, shaken and chilled in a refrigerator at 14h00 by an investigator who took no part in the test sessions or data analysis, but prepared the alphanumeric code identifying the tested drink. At 17h00, the cooled beverages were served in sealed plastic opaque water bottles and consumed using an opaque straw. Participants were instructed to drink the fluid quickly (within 1 ± 0.5 min) 60 min before their test session and not to discuss or compare tastes or to make any assumption about what they had ingested. The interval of 60 min was chosen as being optimal for a complete caffeine absorption [27] and thus enabling the peaking of caffeine concentration [28].

Subjects were supervised by staff to ensure that they drank the entire quantity of fluid, and no exchange of bottles was allowed. The last standardized meal (i.e., lunch) before the beginning of the test session was taken at 13h00. Temperature and relative humidity of the laboratory were similar over the test sessions, with a temperature of around 22 °C and a relative humidity between 45 and 55%. During each test session (from 18h00), resting blood glucose and blood pressures were measured, and the participants completed the POMS questionnaire.

In order to increase body temperature and thus improve the efficiency of the neuromuscular system [22,29,30], a 5-min treadmill warm-up was performed [20] (Figure 1). After that, the reaction time, the handgrip force and the 30-s Wingate tests were performed. RPE scores, blood glucose, and blood pressures were then measured, and the affective load was calculated. To investigate the effects of RB on the acute physiological and psychological responses to exercise, blood glucose and pressure and RPE measures were collected immediately pre- and post-exercise (Figure 1).

### 2.3. Blood Glucose and Blood Pressure Measurements

Blood glucose was measured using the electrochemical sensor Rightest GM260 Blood Glucose Monitoring System (Bionime Corporation, Taichung City, Taiwan). The fingertip was pricked with a lancing device, and a specific test strip was soaked with blood and was inserted into the measuring apparatus, with an estimate appearing within 5 s. Systolic blood pressure was measured by the same physician using a stethoscope (Spengler, Germany) and sphygmomanometer (Spengler, Germany). The intra-class correlation coefficient (ICC) and the standard error of the measurement (SEM) showed good reliability for blood glucose pre- (ICC > 0.72, absolute SEM < 0.03) and post-exercise (ICC > 0.71, absolute SEM < 0.04). Similar results were computed for blood pressure pre- (ICC > 0.72, absolute SEM < 0.32) and post-exercise (ICC > 0.67, absolute SEM < 0.38).

### 2.4. Profile of Mood States (POMS)

The evaluation of mood states was performed using the French language version of the POMS questionnaire. Responses to 65 adjectives (ranging from “Zero” (i.e., not at all) to “Four” (i.e., extremely) assessed immediate mood states in seven dimensions: tension, depression, anger, vigor, fatigue, confusion, and interpersonal relationships. As previous studies (e.g., [31]) utilized only six parameters of the POMS questionnaire due to large variations affecting the dimension of “interpersonal relationships,” this parameter was not included in the analysis. The ICC and SEM showed good reliability for depression (ICC > 0.67, absolute SEM < 1.38), confusion (ICC > 0.68, absolute SEM < 1.39), fatigue (ICC>0.71, absolute SEM < 1.04), vigor (ICC > 0.67, absolute SEM < 1.42), anger (ICC > 0.66, absolute SEM < 1.37), and tension (ICC > 0.68, absolute SEM < 1.26), The ICC and SEM showed, instead, poor reliability for interpersonal relationships (ICC > 0.1, absolute SEM < 1.95).

### 2.5. Rating of Perceived Exertion (RPE) and Affective Load

The original Borg RPE scale rates exertion subjectively during or after physical exercise on a 15-point scale ranging from six (extremely light) to twenty (extremely hard). It was used to calculate the affective load; as suggested by Baron et al. [32], the affective load was obtained as the difference between the perceived exertion (negative affective response) and pleasure scores (positive affective response). For example, with an RPE score of six, the negative affective response is zero and the positive affective response is −14. However, if the RPE score rises to 20, the negative affective response is +14 and the positive affective response is zero. The potential affective load thus ranges from −14 to +14. A negative affective load score indicates the dominance of pleasant affective responses and a positive affective load represents the dominance of unpleasant affective responses [33]. RPE and AL. The ICC and the SEM showed good reliability for RPE and AL (ICC > 0.71, absolute SEM < 0.21).

### 2.6. Reaction Times and Handgrip Strength

A simple visual reaction time test assessed alertness and motor reaction-speed. Subjects responded as quickly as possible to the presentation of a stimulus (the image of a black box) on a computer screen (15” LCD). When this appears, the participant should press the index finger on a computer key. The signal appeared in random order within 1–10-s time intervals. Each participant was allowed ten attempts and the mean reaction time was calculated, using React! V0.9 software.

Handgrip strength was recorded by a dynamometer (T.K.K. 5401; Takei, Tokyo, Japan). The maximal handgrip force was determined for the dominant hand. Participants exerted their maximal strength for 4–5-s. With the hand hanging downwards, the dynamometer was held freely and without support. Three attempts were allowed with 1-min rest intervals, and the largest value was recorded. The ICC and the SEM showed excellent reliability for both reaction time (ICC > 0.89, absolute SEM < 0.14) and handgrip strength (ICC > 0.92, absolute SEM < 0.67) measurements.

### 2.7. Wingate Test

A calibrated mechanically-braked cycle ergometer (Monark 894; Stockholm, Sweden) interfaced with a microcomputer was utilized for the 30-s Wingate test. Subjects pedaled as fast as possible for 30-s against a constant load calculated according to the participant’s body mass (i.e., 8.7%). After maintaining a constant ~60 rpm speed for 4–6-s against minimal resistance, the selected load was applied. The participant sat on the cycle throughout and was strongly encouraged to maximize pedaling rates and to maintain a high speed. Peak and mean power (i.e., the average power output after 30-s) were recorded. The fatigue index was calculated as follows:

Fatigue index (%) = (peak power − minimal power)/peak power × 100(1)

The ICC and the SEM showed excellent reliability for peak power (ICC > 0.98, absolute SEM < 0.21), mean power (ICC > 0.98, absolute SEM < 0.23) and fatigue index (ICC > 0.76, absolute SEM < 1.99).

### 2.8. Statistical Analysis

Results for all parameters are presented as mean ± standard deviation (SD). Data analyses were carried out using the commercial software “Statistical Package for Social Sciences” SPSS v21.0 software (SPSS Inc., Chicago, IL) and Microsoft Excel 2010 (Microsoft Corp., Redmont, WA, USA).

To determine whether two familiarization sessions had been sufficient to remove any learning effects, the intra-class correlation coefficient (ICC) and the standard error of the measurement (SEM) were calculated for all parameters. ICC values over 0.75 were considered as evidence of excellent reproducibility, ICC values between 0.4 and 0.75 were considered as good reproducibility and ICC values less than 0.4 were considered as poor reproducibility.

All parameters met parametric assumptions on the basis of the Shapiro-Wilk’s test. Student’s t-test was used to compare RB and PL and RB with the exception of blood glucose and blood pressures. The effect size (ES) was calculated according to the formula of Glass and magnitudes were interpreted using the Cohen scale: ES < 0.2 was considered as small, ES around 0.5 was considered as medium and ES > 0.8 was considered as large. The mean confidence interval (CI) was determined at 95%.

For blood glucose and blood pressure, a two-way analysis of variance (ANOVA) (2 (Drink) × 2 (Exercise)) was utilized. When a significant main effect or interaction was detected, pair-wise comparisons were assessed using the Bonferroni test in order to ensure protection against multiple comparisons. The Δ-change induced by the drinks (i.e., the difference between PL and RB) was calculated as follow:

Δ-change drink = RB – PL(2)

The Δ-change associated with the exercise bout (i.e., the difference between pre- and post-Wingate) was calculated as follow:

Δ-change exercise = POST – PRE(3)

To assess the relationships between (i) physical and performance and (ii) psychological, physiological, and reaction time parameters, Bland–Altman correlations were used. The significant difference was set at an alpha level of *p* ≤ 0.05 throughout, except in those cases in which multiple comparisons were performed. Exact p-values have been reported and results indicated as “0.000” have been expressed as “<0.0005”.

## 3. Results

### 3.1. Participant Characteristics

**Figure 2:**
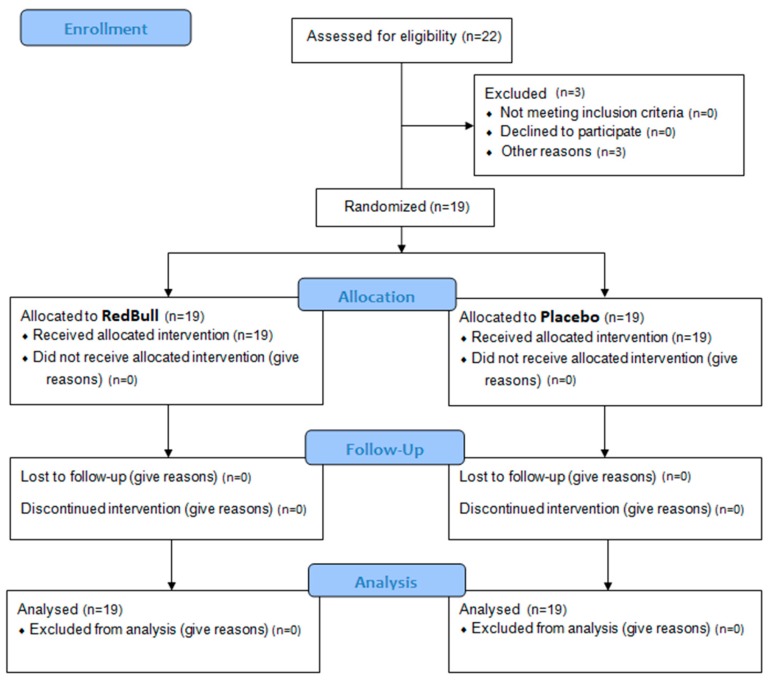
CONSORT flow chart-trial of the study protocol.

Over the study, three participants were unable to complete all test sessions due to muscle pain or injury (Figure 2). Thus, 19 participants (age: 21.2 ± 1.2 years; height: 1.76 ± 0.8 m; body-mass: 76.6 ± 12.6 kg) completed all test sessions (Figure 2).

### 3.2. Physical Performance and Reaction Times

Physical parameters and reaction time recorded during the RB and PL conditions are presented in Table 1.

Statistical analysis showed a significant improvement for peak power (*t* = −2.33; *p* = 0.0250), mean power (*t* = −2.74; *p* = 0.0093), and hand grip force (*t* = −3.21; *p* = 0.0027) between PL and RB and there was a significant reduction in the reaction time (*t* = 5.94; *p* < 0.0005) with RB ingestion as compared to PL, but there was no significant difference of fatigue index between the two drink conditions (*t* = −0.56; *p* = 0.5775).

### 3.3. Blood Glucose and Blood Pressures

**Figure 3:**
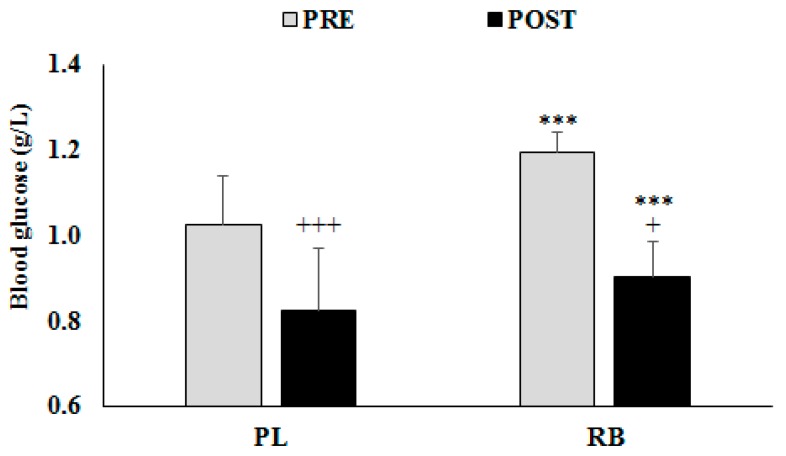
Evolution of blood glucose levels (mean ± SD) from pre- to post-exercise during the placebo (PL) and the Red Bull (RB) sessions. ***: Significant differences compared to PL at *p* < 0.001. +, +++: Significant difference compared to pre-exercise at *p* < 0.05 and *p* < 0.001 respectively.

Blood glucose levels recorded pre- and post-exercise are presented in Figure 3.

There were significant main effects for Drink (F = 36.75; ƞ_p_^2^ = 0.67; *p* < 0.0005) and Exercise (F = 76.94; ƞ_p_^2^ = 0.81; *p* < 0.0005), and the interaction Drink × Exercise was also significant (F = 8.96; ƞ_p_^2^ = 0.33; *p* = 0.0077). Post-hoc testing showed that blood glucose was significantly lower after rather than before exercise in both conditions (*p* < 0.0005 for PL and for RB), with a greater reduction during RB than in the PL condition (Δ-change: −0.29 g/L vs. −0.20 g/L). However, post-hoc testing revealed significant increases of blood glucose with RB in comparison to PL, both pre- (*p* < 0.0005) and post-exercise (*p* = 0.0105), with greater gains before rather than after exercise (Δ-change: 0.17 g/L vs. 0.08 g/L).

**Figure 4:**
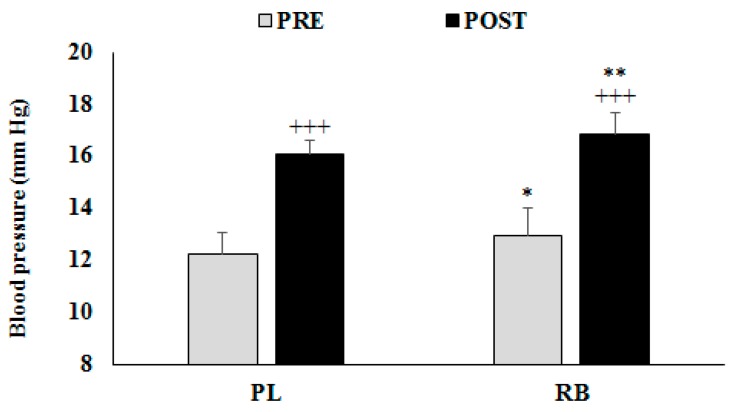
Evolution of blood pressure (Mean ± SD) between pre- and post-exercise during the placebo (PL) and the red bull (RB) sessions. *, **: Significant difference compare to PL at *p* < 0.05 and *p* < 0.01 respectively. +++: Significant difference compared to pre-exercise at *p* < 0.001.

Blood pressures before and after exercise are presented in Figure 4.

There were significant main effects for Drink (F = 34.30; ƞ_p_^2^ = 0.65; p < 0.0005) and Exercise (F = 216.49; ƞ_p_^2^ = 0.92; *p* < 0.0005), but the interaction Drink × Exercise was not significant (F = 0.02; ƞ_p_^2^ = 0.001; *p* = 0.9005). Post-hoc testing showed significantly higher values of blood pressure after than before exercise in both conditions (*p* < 0.0005). Moreover, post-hoc tests revealed significant greater blood pressure with RB in comparison to PL at both pre- (*p* = 0.0120) and post-exercise (*p* = 0.0080).

### 3.4. Ratings of Perceived Exertion (RPE), Affective Load and Profile of Mood States (POMS)

POMS parameters, affective load and RPE during PL and RB conditions are presented in Table 2.

Although no significant difference between the two drinks conditions was reported for depression (*t* = 1.07; *p* = 0.2952) and anxiety (*t* = 1.94; *p* = 0.0675), RB was associated with a significant reduction in scores for confusion (*t* = 2.32; *p* = 0.0322), fatigue (*t* = 2.34; *p* = 0.0305), anger (*t* = 2.43; *p* = 0.0258), RPE (*t* = 4.30; *p* = 0.0001), and affective load (*t* = 4.77; *p* = 0.0001). In contrast, vigor increased significantly (*t* = −2.63; *p* = 0.0167) with RB.

### 3.5. Correlations between the Recorded Parameters

Peak power was significantly correlated with RPE (*r* = −0.48; *p* = 0.0322), affective load (*r* = −0.48; *p* = 0.0322), pre- (*r* = 0.59; *p* < 0.01) and post-exercise (*r* = 0.65; *p* = 0.0019) levels of blood glucose, and scores for fatigue (*r* = −0.50; *p* = 0.0230) and anxiety (*r* = −0.50; *p* = 0.0244). Mean power was also significantly correlated with RPE (*r* = −0.64; *p* = 0.0023), affective load (*r* = −0.64; *p* = 0.0023), pre- (r = 0.68; *p* = 0.0009) and post-exercise (*r* = 0.69; *p* = 0.0007) blood glucose levels, pre-exercise blood pressure (*r* = −0.50; *p* = 0.0233) and scores for fatigue (*r* = −0.55; *p* = 0.0113) and anger (*r* = −0.54; *p* = 0.0148). However, no significant correlations were observed between fatigue index and blood glucose, blood pressure POMS scores, affective load, or RPE. Handgrip force was significantly correlated with RPE (*r* = −0.60 *p* = 0.0049), affective load (*r* = −0.60; *p* = 0.0049), pre- (*r* = 0.78; *p* < 0.0005) and post-exercise (*r* = 0.57; *p* = 0.0082) blood glucose and pre- (*r* = 0.62; *p* = 0.0037) and post-exercise (r = 0.54; *p* = 0.0139) blood pressures. Reaction time was significantly correlated with RPE (*r* = 0.72; *p* < 0.0005), affective load (*r* = 0.72; *p* < 0.0005), pre- (*r* = 0.57; *p* = 0.0080) and post-exercise (*r* = 0.48; *p* = 0.0290) blood glucose, pre- (*r* = −0.73; *p* < 0.0005) and post-exercise (*r* = −0.72; *p* < 0.0005) blood pressures and POMS scores for confusion (*r* = 0.46; *p* = 0.0387), vigor (*r* = −0.62; *p* = 0.0035), and anger (*r* = 0.46; *p* = 0.0393).

## 4. Discussion

The main findings from the present study were that RB increases peak power (+0.93 W·kg^−^^1^) and mean power (+0.87 W·kg^−^^1^) during the 30-s Wingate test, and handgrip force (+2.69 kg), also speeding the reaction time (−0.08 s). Additionally, physiological responses to exercise (i.e., blood glucose and blood pressure) are increased and the RB increases vigor with reduction of ratings for depression, confusion, fatigue, anger, anxiety, RPE, and affective load.

In agreement with the present results, Alford et al. [11] reported that RB had a positive effect on short-term maximal performance during the Wingate test. Forbes et al. [34] also found that RB tended to a positive (but not significant effect) on peak and mean power during three consecutive Wingate tests. The latter authors also reported significant increases in total repetitions over three sets of bench press exercises.

In the present study, increases of peak and mean power during the 30-s Wingate test were significantly correlated with decreases in RPE, affective load, and scores for fatigue, anxiety (for peak power only), and anger (for mean power only). Increases in handgrip force were also related to decreases of RPE and affective load. A previous study also reported that the handgrip force was greater after caffeinated-ED than after PL [7,35]. In agreement with the present study’ results, Hahn et al. [8] reported significant reductions in fatigue scores when performing a repeated-sprint exercise. However, they did not show any improvement in performance during the repeated sprinting. From the present results, the increases in short-term maximal performances induced by RB could be explained by a reduced perception of exertion and fatigue. Increases are also related to a reduction of affective load, a change of perceptions in that part of the brain responsible for pacing strategy during physical exercise.

Pacing regulates energy expenditures during exercise. Better short-term maximal performance after RB ingestion reflects higher energy expenditures, as shown by the higher pre- and post-exercise blood glucose concentrations during the RB session and by the greater decreases of blood sugar from before to after exercise (−0.29 g/L vs. −0.20 g/L in PL).

Lim et al. [36] showed that in people who do not normally consume caffeine, taurine ingestion is detrimental to maximal voluntary muscle power and both maximal isometric and isokinetic peak torque, whereas taurine ingestion in caffeine-deprived caffeine consumers improves maximal voluntary muscle power but has no effect on other aspects of contractile performance.

Graham et al. [37] showed that the beneficial effects of caffeine ingestion on short-term maximal performance were related to muscle fat oxidation and better glycogen sparing capacity. A recent meta-analysis by Grgic [38] and by Grgic et al. [39] concluded that caffeine ingestion may increase both peak and mean power output during the Wingate test. In an umbrella review of 21 published meta-analyses, Grgic et al. [40] concluded that caffeine ingestion improved a broad range of exercise performance measures such as muscle strength, muscle endurance, anaerobic power, and aerobic endurance. Mechanisms explaining such findings include an increased Ca^2+^ release from the sarcoplasmic reticulum, which may lead to an increase in tetanic tension, and the alterations that caffeine might have on the neuromuscular transmission [41]. In an animal study, it has been shown that caffeine may enhance Ca^2+^ release from the sarcoplasmic reticulum and improve motor unit recruitment by inhibiting the action of adenosine on the central nervous system [42]. Glucose is an important metabolic substrate responsible for most of the energy release during anaerobic exercise. Thus, the pre- and post-exercise increases of blood glucose could, in part, explain the improvement of short-term maximal performance.

In support of these hypotheses, the present results demonstrated a significant correlation between peak and mean power during the 30-s Wingate test and the pre- and post-exercise glucose. Also, it has been reported that glucose increases are related to an improvement in cognitive performance [43]. In this context, the present study showed significant correlations between pre- and post-exercise blood glucose and reaction time. Alford et al. [11] also reported significant improvements in choice reaction time, memory, and concentration (i.e., the number of correct cancellations) after RB ingestion compared to the PL condition. These authors concluded that RB ingestion improved alertness. This same conclusion is supported by Mets et al. [44], who showed that RB ingestion improved driving performance and reduced driver sleepiness. The present study indicated a significant correlation between peak and mean power during the Wingate test and negative components of mood state (i.e., anxiety and anger). Lara et al. [45] also reported a significant improvement in the short-term maximal performance of swimmers and a significant reduction in anxiety scores after ED consumption.

The present study demonstrated a significant correlation between (i) reaction time and (ii) positive (i.e., vigor) and negative (i.e., confusion and anger) components of mood state. Therefore, the enhancement of reaction time could be explained in part by a reduction of confusion and anger and an improvement of vigor. These findings are supported by Wesnes et al. [15] who suggested that cognitive performance increased with the improvement of positive and a reduction of negative components of mood state. On the other hand, as previously reported by Del Coso et al. [4,5] and Abian-Vicen et al. [6] some subjects do not seem to respond to the ergogenic effects of caffeine-containing ED. In the present study, four participants could be classified as non- responders in terms of their performance on the Wingate test; although their performance of the handgrip and reaction time tests did improve after RB ingestion compared to the PL.

### Limitations

One limitation when interpreting this research is that the commercially-prepared ED evaluated contained several potentially ergogenic ingredients, including compounds such as caffeine, carbohydrates, and taurine while the PL control drink did not include these substances. Therefore, it was not possible to identify the specific influence of any one of these several active ingredients on performance. Future studies should focus on the specific influence of individual active ingredients.

Another limitation inherent to the present study is that the participants were not regular caffeine users and, then, results could not be generalized to people who do regularly consume caffeine. Also, the fact that no-baseline (i.e., before RB or PL consumption) measurement was performed represents another shortcoming of the present investigation.

No immediate adverse effects were seen from the RB, but a more deliberate search for negative consequences of caffeine ingestion, such as an increase of speed at the expense of skills, would seem justified. Another limitation of the present study is that enrolled subjects were all male. Future studies using female or mixed-gender samples are warranted.

Further, the effectiveness of subject blinding was not tested by post-study debriefing. This is of some importance, because outcomes may be influenced if a participant recognizes that one of the beverages provided contains caffeine [46]. Tallis et al. [47] underlined that the psychological effects of “expectancy” and “belief” could have a significant impact on performance. Therefore, future studies should use a double-blind design and assess the effectiveness of the blinding.

## 5. Conclusions

The present study has demonstrated that cognitive (i.e., reaction time) and short-term maximal (i.e., handgrip and Wingate) performances are improved after RB ingestion. Further, ingestion of this ED increases physiological responses to the 30-s Wingate test, with increases of pre- and post-exercise blood glucose and blood pressures. Further RB consumption reduces negative effects on mood state (i.e., decreased scores for depression, confusion, fatigue, anger, and anxiety) and enhances the positive components of mood state (i.e., vigor), with favorable changes of RPE and affective load, thus leading to improvements in physical performance.

Gains of physical performance after RB consumption reflect changes in blood glucose and blood pressure. Cognitive gains (i.e., a speeding of reaction time) are related to both psychological (i.e., a reduction of confusion, anger, and RPE and an increase of vigor) and physiological responses (i.e., changes in blood glucose and blood pressure) ergogenic changes.

## Figures and Tables

**Table 1:** Confidence intervals (CI), Δ-change and mean and standard deviations (SD) of peak and mean power and the fatigue index registered during the Wingate test, handgrip force, and reaction times recorded during the Red Bull (RB) and placebo (PL) conditions.

Parameters	PL		RB		Δ-Change
	Mean ± SD	CI	Mean ± SD	CI	
**Peak power (W·kg^−1^)**	10.5 ± 1.5	9.8–11.2	11.4 ± 0.9	11.0–11.9	0.93 *
**Mean power (W·kg^−1^)**	8.1 ± 1.0	7.65–8.63	9.01 ± 0.92	8.56–9.46	0.87 **
**Fatigue index (%)**	47.9 ± 8.1	44.0–51.8	49.1 ± 4.8	46.8–51.4	1.21
**Hand grip force (kg)**	55.5 ± 2.7	54.18–57.03	58.2 ± 2.4	56.8–59.4	2.69 **
**Reaction time (s)**	0.36 ± 0.05	0.34–0.39	0.28 ± 0.02	0.27-0.29	−0.08 ***

*, **, ***: Significant difference between RB and PL at *p* < 0.05, *p* < 0.01 and *p* < 0.001 respectively.

**Table 2:** Confidence interval (CI), Δ-change and mean and standard deviation (SD) for individual Profile of Mood State parameters (i.e., depression, confusion, fatigue, vigor, anger, and anxiety) and ratings of perceived exertion (RPE) recorded during the red bull (RB) and placebo (PL) conditions.

POMS Parameter/RPE	PL		BE		Δ-Change (AU) Induced by RB
	Mean ± SD	CI	Mean ± SD	CI	
Depression (AU)	6.1 ± 5.1	3.6–8.5	4.8 ± 2.2	3.8–5.9	−1.2
Confusion (AU)	7.3 ± 6.0	4.4–10.2	4.2 ± 2.0	3.2–5.1	−3.1 *
Fatigue (AU)	13.8 ± 2.1	12.9–14.8	12.7 ± 2.1	11.7–13.8	−1.1 *
Vigor (AU)	14.7 ± 5.8	12.0–17.5	18.2 ± 4.2	16.1–20.2	3.4 *
Anger (AU)	4.8 ± 3.2	3.2–6.3	3.4 ± 3.2	1.9–5.0	−14.*
Anxiety (AU)	10.8 ± 5.3	8.2–13.3	8.3 ± 2.9	6.9–9.7	−2.5
RPE (AU)	17.5 ± 1.3	16.9–18.2	15.9 ± 1.1	15.4–16.4	−17 ***
Affective load (AU)	9.1 ± 2.6	7.8–10.3	5.8 ± 2.1	4.8–6.8	−3.3 ***

*, ***: Significant difference between RB and PL at *p* < 0.05 and *p* < 0.001 respectively.
